# Legal Notes for Institutional Workers

**Published:** 1920-04-17

**Authors:** 


### LEGAL NOTES FOR INSTITUTIONAL WORKERS.

Medical Evidence in a Compensation Case.—*Lancaster v. Blackwell Colliery Coy., Ltd.* (146 L.T.J. 21), was a case under the workmen's compensation into which medical evidence appears to have entered more than into any case which, so far as we are aware, has yet occurred under that statute. The point was whether the death of the workman in question was due to "personal injury by accident arising out of and in the course of his employment." The workman died from internal strangulated hernia. Part of the bowel had passed through an abnormal cavity which existed in the mesentery of the colon; and the bowel, being retained in that position, by constriction, caused death. The widow of the workman, as his dependent, accordingly made a claim for compensation against the employers under the Workmen's Compensation Act, 1906, which was followed in due course by a request for arbitration. The medical evidence, as it appeared to the County Court judge, showed that the cavity in the mesentery was not attributable in any way to the workman's employment. The intrusion of the bowel into the cavity might have been caused in many ways, as the evidence, the County Court judge considered, plainly showed. But only one of such causes was known to have been present at the time when the workman's symptoms exhibited that the trouble which ended in his death had begun. That cause was the violent exertion to which his work put him. And his death was attributed to that cause, according to the view taken by the County Court judge, and he decided that the occurrence was, therefore, an accident arising "in the course of his employment" and likewise "out of it," within the meaning of Section 1 of the Act. With regard to the cause of death, the only evidence that diminished at all the strong probability of the connection between the workman's exertions and the intrusion of the bowel was that of the doctors, who said that a gangrenous condition might be expected to set in within from a few hours to three days. But this evidence was given with expressions of unwillingness to be tied to any time and statements that it depended on the closeness of the stricture. The County Court judge accordingly made his award in favour of the workman's dependant. The employers appealed. It was held that on the medical evidence no accident to the workman could have happened at the alleged time; and that there was no evidence that his injuries resulted therefrom rather than from internal causes. Held, therefore, that the County Court judge was wrong in law in deciding that the death of the workman was caused by "personal injury by accident arising out of and in the course of his employment."

